# The Thermal Behavior of Lyocell Fibers Containing Bis(trimethylsilyl)acetylene

**DOI:** 10.3390/polym13040537

**Published:** 2021-02-11

**Authors:** Igor Makarov, Markel Vinogradov, Maria Mironova, Georgy Shandryuk, Yaroslav Golubev, Anna Berkovich

**Affiliations:** 1 A.V. Topchiev Institute of Petrochemical Synthesis, Russian Academy of Sciences, 29 Leninsky prospect, 119991 Moscow, Russia; vin1989@ips.ac.ru (M.V.); gosha@ips.ac.ru (G.S.); 2 Chemical Department, M.V. Lomonosov Moscow State University, 1 Leninskiye Gory, 119991 Moscow, Russia; GolubevYV@vms.chem.msu.ru (Y.G.); annber@vms.chem.msu.ru (A.B.)

**Keywords:** cellulose, bis(trimethylsilyl)acetylene, lyocell fibers, thermal properties, coefficient of thermal expansion

## Abstract

This study focuses on the preparation of carbon fiber precursors from solutions of cellulose in *N*-methylmorpholine-*N*-oxide with the addition of bis(trimethylsilyl)acetylene, studying their structural features and evaluating thermal behavior. The introduction of a silicon-containing additive into cellulose leads to an increase in the carbon yield during carbonization of composite precursors. The type of the observed peaks on the differential scanning calorimetry (DSC) curves cardinally changes from endo peaks intrinsic for cellulose fibers to the combination of endo and exo peaks for composite fibers. For the first time, coefficient of thermal expansion (CTE) values were obtained for Lyocell fibers and composite fibers with bis(trimethylsilyl)acetylene (BTMSA). The study of the dependence of linear dimensions of the heat treatment fibers on temperature made it possible to determine the relation between thermal expansion coefficients of carbonized fibers and thermogravimetric curves, as well as to reveal the relationship between fiber shrinkage and BTMSA bis(trimethylsilyl)acetylene content. Carbon fibers from composite precursors are obtained at a processing temperature of 1200 °C. A study of the structure of carbon fibers by X-ray diffraction, Raman spectroscopy, and transmission electron microscopy made it possible to determine the amorphous structure of the fibers obtained.

## 1. Introduction

Carbon fibers (CFs), as an important class of heat-resistant fibers [1], have been intensively studied due to their excellent properties, such as high strength, heat resistance, low density, and chemical stability [2,3,4]. The main consumers of CFs are the aerospace industry, the automotive industry, sports equipment, and medicine [5,6,7].

The production of cellulose-based carbon materials is a current task, despite the fact that high-strength carbon fibers (CFs) are produced on an industrial scale from precursors based on polyacrylonitrile and pitch [8]. The relevance of the problem lies in obtaining CFs not only with the required mechanical characteristics, but also with a necessary coefficient of thermal expansion (CTE), which is known to be closely related to the structure of such fibers [9]. An important task remaining is a solution to the problem of replacing petroleum products with renewable plant sources of polymers that are processed into CFs. The importance of CTE values is related to the performance properties of the resulting composite materials where carbon fibers are used as a functional additive. Close values of the CTE of the matrix and the filler exclude the formation of defects, and as a consequence, give the best (stable) mechanical characteristics with a change in the temperature of the composite material [10,11].

The correct choice of a type of precursor and a process of its heat treatment allows for a necessary structure to be formed, and this directly affects CTE values of carbon fibers. Pradere and Sauder [12] reported that the largest values of the CTE in a longitudinal direction and the smallest in a transverse direction were observed for pitch-based carbon fibers (unoriented samples). The formation of an anisotropic structure in the sample leads to a change to opposite values.

Unlike polyacrylonitrile (PAN) precursors, during carbonization and further graphitization of cellulose fibers the interplanar spacing d_002_ and the mutual arrangement of crystalline layers (strips of CF) are similar to graphite [13]. Consequently, there will be differences in the CTE values of carbon fibers obtained from such precursors.

Due to the specific structure of cellulose, the range of its direct solvents is limited [14]. Therefore, at the present moment, cellulose fibers, including technical yarns (cord), which are used as precursors of CF, are produced on an industrial scale in the viscose and *N*-methylmorpholine-*N*-oxide (NMMO) processes only [15]. If the first process is dominated by the volume of fibers produced, the second is characterized by smaller amounts of harmful emissions and higher energy efficiency [16].

According to the accepted classification of the international committee BISFA (International Bureau of Standardization of Man-Made Fibres (Brussels, Belgium)), fibers formed from cellulose solutions in NMMO received the general name Lyocell [17].

A decrease in the water content of NMMO increases its dissolving capacity with respect to cellulose. Using the direct solvent NMMO with water content of about 8% makes it possible to obtain Lyocell fibers from concentrated cellulose solutions (up to 18%) [18]. The use of NMMO with a low water content is associated with high melting points of NMMO and obtaining solutions [19]. This imposes restrictions on the possible ways of processing solutions into fibers. Therefore, the dry–wet jet method of spinning fibers from cellulose solutions in NMMO is the main one used [20]. Water or aqueous NMMO solutions are often used as a precipitant [21].

The maximum possible carbon yield obtained during pyrolysis of cellulose does not exceed 44.4 wt% [22]; however, in reality, these values rarely exceed 30% and depend on pre-treatment and thermolysis regimes [23]. An accumulated experience of processing cellulosic precursors into carbon fibers has shown that it is impossible to obtain high-quality CFs and increase carbon yield without the flame retardants and pyrolysis catalysts, which are able to influence the course of the thermal reaction used [24,25,26,27]. Production of carbon fibers from cellulosic precursors using ammonium compounds became a trend. Without concentrating on disadvantages of using such flame retardants and pyrolysis catalysts, we note that the use of silicon-containing compounds is proposed as an alternative. There are two main ways of using these compounds: impregnating the fibers with their solutions [28,29] and introducing them into dope as an additive [30].

In our previous work [31,32,33,34], advantages of the second method were revealed in comparison to fiber impregnation. It was also found that the introduction of organosilicon compounds into the cellulose matrix makes it possible to achieve a better distribution of the additive in the volume of cellulose fibers, to increase the yield on carbon, and to obtain composite carbon–silicon carbide composite fibers. The thermal behavior of the composite precursors is correlated with their structure and the chemical nature of the additive. Namely, the switch from tetraethoxysilane (TEOS) with a high C/O ratio to vinyltriethoxysilane (VTEOS) that has in its structure Si–C and double bonds leads to reducing the activation energy of pyrolysis and decreasing the carbon yield.

The use of oxygen-free silicon-containing compounds is likely to reduce the mass loss during pyrolysis, where carbon loss is known to be accompanied by the formation of volatile CO and CO_2_. Therefore, the task of this work was to obtain composite precursors based on cellulose and bis(trimethylsilyl)acetylene through solid-phase solutions in NMMO, and to study their structure and thermal behavior, as well as to determine the chemical composition and structure of the resulting carbon fibers.

## 2. Materials and Methods

### 2.1. Materials

Sulfate powder cellulose with degree of polymerization DP = 600, a moisture content of 7–8%, and a mass content in the dry residue of alpha cellulose of 94% was provided by Baikal Pulp and Paper Mill (Baykalsk, Russia). The silicon-containing additives (bis(trimethylsilyl)acetylene (BTMSA) (ID 24851275)) were obtained from Sigma-Aldrich (St. Louis, MO, USA). Direct solvent of cellulose *N*-methylmorpholine-*N*-oxide (NMMO) with *T_m_* = 120 °C (H_2_O < 10%) was supplied by Demochem (Shanghai, China). Solutions containing 18% cellulose with and without BTMSA in NMMO were prepared through a solid-phase activation method from mixtures of cellulose/BTMSA with content: 0, 5, and 10 wt% BTMSA (further in the text the designation of fiber samples is Si-0, Si-5, and Si-10, respectively) [35]. Propyl gallate (0.5 wt%) from Sigma-Aldrich was used to suppress thermo-oxidative degradation of the solvent.

### 2.2. Fiber Spinning

The fibers were formed by the dry–wet jet method on a Rheoscope 1000 capillary viscometer (CEAST, Italy) equipped with a fiber winding system (capillary diameter, d, 1 mm; length, l, 40 mm; l/d = 40). The size of the air gap between the capillary and the aqueous precipitation bath is 10 cm. After the precipitation bath, the fibers were further washed with water until NMMO was completely removed. At the final stage, the fibers were dried at ambient conditions in a free state.

### 2.3. Structure Characterization

The structure of cellulose and composite fibers was investigated by X-ray diffractometry using a Rigaku Rotaflex-RC (Rigaku Corporation, Tokyo, Japan) unit equipped with a rotating copper anode (30 kV–100 mA source operating mode, characteristic wavelength of CuK_α_ radiation λ = 0.15418 nm), a horizontal D-Max/B goniometer, and a scintillation detector. A nickel filter was used to absorb CuK_β_ radiation. X-ray experiments were performed at room conditions in a “Bragg–Brentano” transmission mode geometry in the continuous θ–2θ scanning mode (angular range of 2θ, 2.5–50°; scanning step, 0.04°). To obtain the diffractograms of the fibers, parallel bundles of their fragments (around 100 pieces) were used in vertical or horizontal directions relative to the goniometer axis.

The Raman scattering measurements were performed in a Raman spectrometer (Horiba LabRAM HR Evolution, Kyoto, Japan) equipped with a 532 nm laser excitation wavelength at room temperature. The Raman spectrometer was operated in the continuous scanning mode.

### 2.4. Mechanical Properties

Specimens of composite fibers with BTMSA were tested using an Instron universal tensile machine (Instron, model 1122, Norwood, MA, USA) supplied with pneumatic clamps. The fiber diameters were measured with an optical microscope (Biomed-6PO, Moscow, Russia). An extension rate of 10 mm min^−1^ and a working length of 10 mm were used for all specimens.

### 2.5. Scanning Electron Microscopy (SEM)

Micrographs of the surface and cross-section of cellulose and composite fibers were obtained on a scanning electron microscope (JSM U-3, JEOL, Tokyo, Japan). Cellulose fibers, unlike carbon fibers derived from them, are less brittle. That is why, in order to obtain SEM micrographs, cellulosic precursors were previously placed in an epoxy rubber matrix, and then after they were cured, a cross-section was made using a microtome cutter. The cross-sections of the carbon fibers were obtained by a simple mechanical action.

Structures, morphologies, and elemental compositions of fibers produced at 1200 °C were studied on an Osiris transmission electron microscope (FEI, Hillsboro, OR, USA) at an accelerating voltage of 200 kV in transmission electron microscopy, high-resolution electron microscopy, and scanning-transmission electron microscopy modes (using an energy-dispersive X-ray spectrometer).

### 2.6. Thermal Properties

The thermal behavior was examined on a thermogravimetric analyzer and differential scanning calorimeter combined instrument (TGA/DSC1, Mettler Toledo, Greifensee, Switzerland). The measurements were carried out in the temperature range from 30 °C to 1000 °C at a heating rate of 10 °C min^−1^. The flow rate of the argon reaction gas was 10 mL min^−1^. Alumina crucibles of 70 μL were used. Thermomechanical analysis (TMA) was performed on a TMA 402 F1 Hyperion instrument (Netzsch, Selb, Germany) (SiC furnace, Al_2_O_3_ holder). The measurement mode was tension. The fibers of the precursor sample were fixed in clamps with a total sample diameter of 1 mm. The distance between the clamping plates was 20 mm for the first measurement and 10 mm for the next one. To prepare tensioned specimens, an alignment jig was used to fix the distance between corundum clamps of 20 mm and 10 mm, respectively. At the first stage, the samples were heated in an Ar atmosphere from 25 °C to 500 °C at a rate of 10 °C min^−1^ with a total flow of 70 mL min^−1^. The applied tensile force during heating was constant and equal to 100 mN. At the second stage, the samples were reheated in an Ar atmosphere from 25 °C to 1200 °C at a rate of 10 °C min^−1^ with a total flow of 70 mL min^−1^. The applied tensile force during heating was constant and equal to 50 mN.

## 3. Results

### 3.1. Structure of Cellulose and Composite Fibers

**Figure 1:**
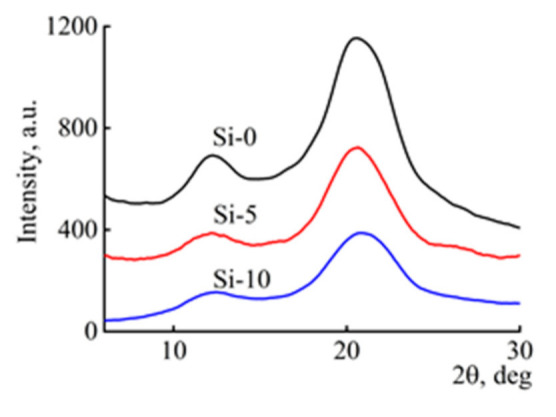
Equatorial diffractograms of Si-0, Si-5, and Si-10 fibers.

Introduction of silyl-substituted acetylene into cellulose solutions does not practically affect the rheological behavior of the filled solutions; i.e., it did not require a change to the spinning procedure. Therefore, just as in the case of cellulosic solutions, the solutions with BTMSA were formed by the dry-jet wet method. The diffractograms of the fibers obtained are shown in Figure 1.

In the process of cellulose solution preparation and regeneration, the system of hydrogen bond was broken, resulting in the destruction of the cellulose I structure, which made the crystal structure of cellulose I turn into cellulose II. The introduction of BTMSA into the cellulose matrix does not lead to a change in the angular position of the basal reflections of cellulose 2θ = 12.3° and 20.6°, which corresponds to the interplanar distance of 3.61 Å and 2.19 Å, respectively, and belongs to the cellulose polymorphic form II [36]. An increase in the content of BTMSA in the fibers results in a change in the intensity of the main reflections and the cellulose crystallinity.

### 3.2. Morphology of Cellulose and Composite Fibers

**Figure 2:**
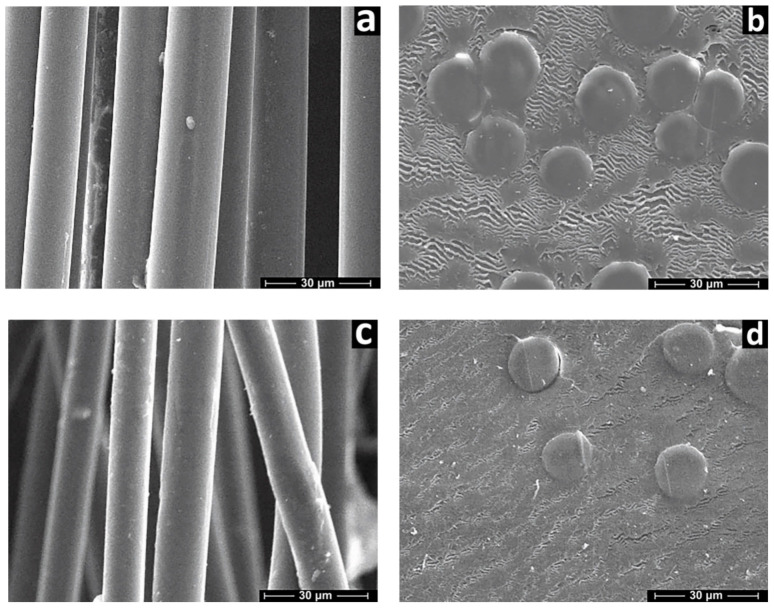
Microphotographs of Si-0 (**a**,**b**) and Si-5 (**c**,**d**) fibers (**a**,**c**—surface; **b**,**d**—cross-section).

The SEM images of cellulose and composite fiber surfaces and cross-sections are shown in Figure 2.

For all spun fibers, a circular shape and regular size were observed along the fiber axis, consistent with the cross-section morphology given by Rous [37]. The surface of the fibers is smooth and without visible anomalies. Pictures (b and d) show the cross-sections of the cellulose and composite fibers, respectively. Voids or defects are missing in the cross-sections. The cellulose and composite fibers show a relatively dense structure. The absence of delamination indicates acceptable adhesion of the components during coagulation. This suggests that the process of coagulation of cellulose and silicon-filled solutions proceeds according to the same mechanism.

### 3.3. Mechanical Properties

Structural changes are caused by a composite additive and its effect on the mechanical characteristics of the fibers (Table 1).

Composite fibers compared with cellulose are characterized by lower values of strength and elongation at break. The fibers containing BTMSA show a decreased tensile strength, especially when the BTMSA content is 10 wt%. The modified fibers also show lower values of elongation at break than those of unmodified fibers. For Si-5 or Si-10, elongation is 7%, respectively. On the other hand, all the modified fibers show a higher modulus in comparison with unmodified fibers. The highest values of modulus are observed for Si-5. In general, the changes observed in the values of mechanical characteristics are not critical and it is possible to use the obtained composite fibers as precursors for CFs.

### 3.4. Thermal Behavior

**Figure 3:**
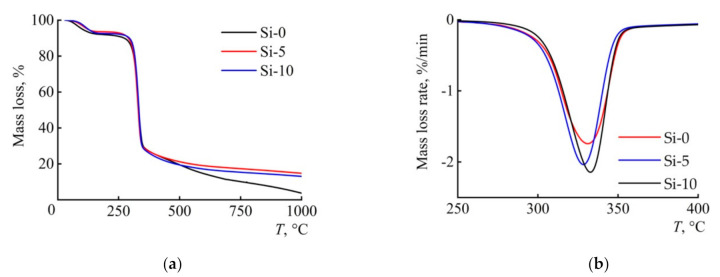
Thermogravimetric curves (TG) (**a**) and differential curves of the mass loss rate DTG (**b**) for Si-0, Si-5, and Si-10 fibers. Atmosphere: argon 10 mL min^−1^. Linear heating rate of 10 °C min^−1^.

**Figure 4:**
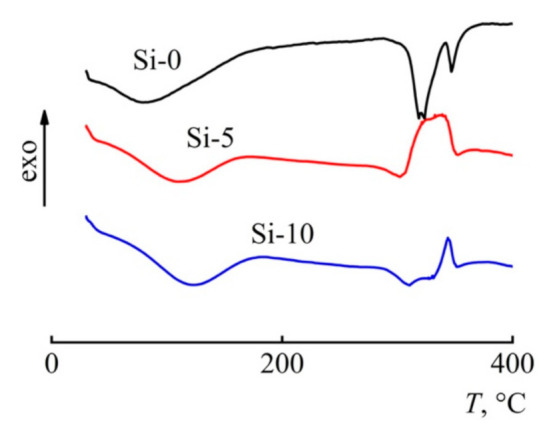
DSC curves for Si-0, Si-5, and Si-10 fibers. Atmosphere: argon 10 mL min^−1^. Linear heating rate of 10 °C min^−1^.

It is known that the thermal behavior of fibers depends on both the structure of the precursor [38,39] and the chemical nature of the additive used [40]. Therefore, to determine the effect of BTMSA on pyrolytic mechanisms and the carbon yield, the thermal behavior of the fibers was studied by differential scanning calorimetry (DSC) and thermal gravimetric analysis (TGA) (Figure 3 and Figure 4).

The course of thermogravimetric curves for cellulose and composite fibers has a traditional appearance and includes areas for release of the adsorbed moisture, dehydration, and depolymerization and a high-temperature region with lower rates of mass loss [41]. The addition of 5% BTMSA into cellulose leads to a significant increase in the values of the carbon yield. A further increase in the additive content up to 10% does not provide an increase in the carbon yield. Table 2 presents the main results of the TG studies.

In the study, the single heating rate kinetic method was applied. Generally, this method is not recommended by the International Confederation for Thermal Analysis and Calorimetry Kinetic Committee [43,44,45]—but as an express method for evaluating *E_a_*, it is convenient and allows one to get the first ideas about the activation energy of the pyrolysis process. Comparing onset temperatures of degradation revealed that cellulose fibers had the same thermal stability as compared with the composite fibers. At the same time, the activation energy of pyrolysis for composite fibers is reduced by 30%. With increasing concentration of BTMSA, an increase in the maximum rate of mass loss is observed.

Along with the TGA studies, DSC curves for cellulose and composite fibers were obtained. Endo-effects that appeared in the process of pyrolysis of cellulose fibers are converted into a combination of endo- and exo-effects. The mechanism for changing the nature of the peaks is not yet completely clear; however, it can be assumed that the nature of the additive contributes to the process along with structural factors.

**Figure 5:**
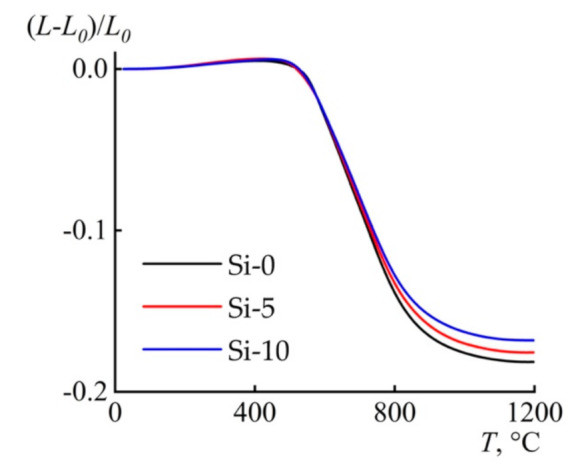
Thermomechanical analysis (TMA) curve for of Si-0, Si-5, and Si-10. Atmosphere: argon 70 mL min^−1^. Linear heating rate of 10 °C min^−1^. *L*—current sample size; *L_0_*—initial sample size.

The difference in weight loss for fibers containing BTMSA is formed during the thermal decomposition stage and is fixed at a temperature of about 500 °C (Figure 5). TMA of fibers preliminarily subjected to heating to 500 °C in an argon atmosphere showed a difference in CTE of fibers in the temperature range of 25–400 °C and absolute values of expansion of the samples along the drawing direction (Table 3). The largest value corresponds to the sample containing 5% BTMSA, and the smallest value to that of the cellulose fibers. The increase in thermal expansion can be explained by the appearance of additional regions of ordering, which is in agreement with the pattern of thermogravimetric curves. An increase in BTMSA content reduces fiber shrinkage, which can be observed by the divergence of thermomechanical curves in the temperature range 620–1200 °C. The observed phenomenon is possibly related to the melting of SiO_2_ silicates and other products formed as a result of joint heating of cellulose and BTMSA.

### 3.5. Morphology and Structure of Carbonized Fibers

**Figure 6:**
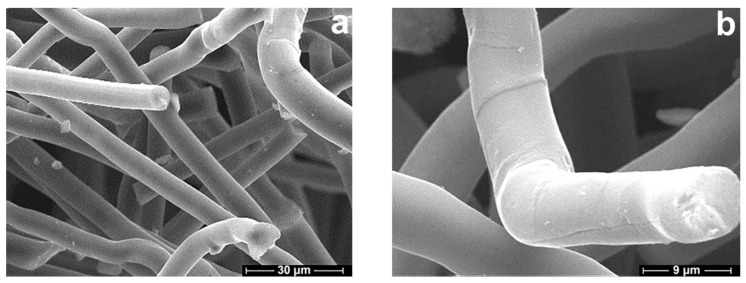
SEM micrographs of the surface (**a**) and cross-section (**b**) morphology of carbon fibers, derived from Si-5 fibers at 1200 °C.

Practice shows that the morphology of carbonized fibers directly depends on the morphology of precursors [46]. That is why a round cross-section, few physical defects, and fine denier of precursors give hope for similar characteristics in carbon fibers. After carbonization of composite precursors, the average fiber diameter decreases from 12 μm to 6 μm (Figure 6).

From a comparison of the morphology of precursors (Figure 2) and carbonized fibers, it can be concluded that the main geometric characteristics of the fibers are preserved. The surface of the fibers is smooth with no observable defects. The thickness of the fibers is maintained along the fiber axis.

**Figure 7:**
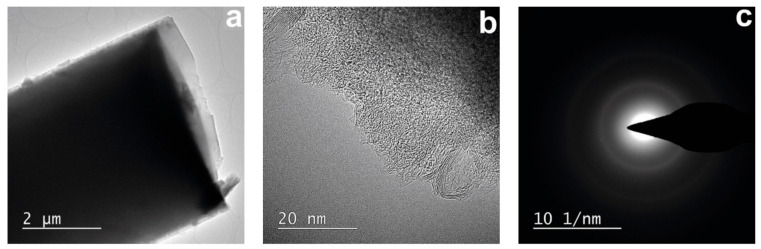
TEM images (**a**,**b**) of carbon fibers obtained from precursors Si-10 by heat treatment up to 1200 °C and selected area electron diffraction (SAED) patterns of amorphous area of carbon fibers (CFs) (**c**).

It is possible to trace the supramolecular organization of carbon in the obtained CFs by using the TEM method (Figure 7).

The surface area and the cross-section of the fibers (Figure 7a,b) are “transparent” for the electron beam, which makes it possible to scan the fibers. Comparison of the fibers’ surface and cross-section morphology revealed a predominantly isotropic structure for the sample, which corresponds to diffractograms with diffuse rings (Figure 7c). A study of the cross-section with a higher magnification revealed two types of structures (Figure 7b). The first type is composed of randomly “intertwined” carbon layers, and the second is ordered domains consisting of parallel layers. The distance between the layers of the domains (d_002_) varies over a wide range, though not reaching values characteristic of graphite (0.3354) [47].

**Figure 8:**
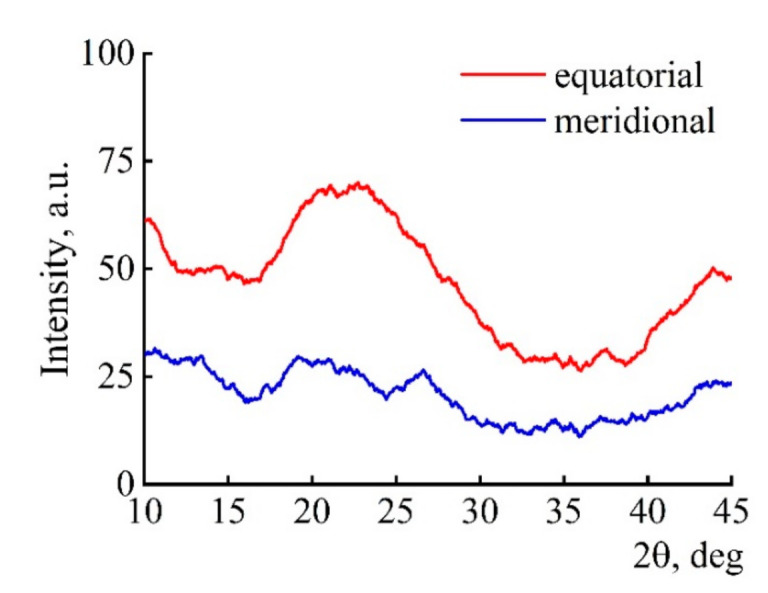
X-ray diffraction patterns of composite fibers (Si-10).

The diffractograms of the fibers (Figure 8) revealed peaks in the range of 2θ = 22.5° (002) and 43.9° (100). The d_002_ values obtained by the TEM method and calculated using the Bragg formula have a common size range. The broad peak (002) observed in the diffraction pattern can be attributed to the amorphous carbon phase [48].

**Figure 9:**
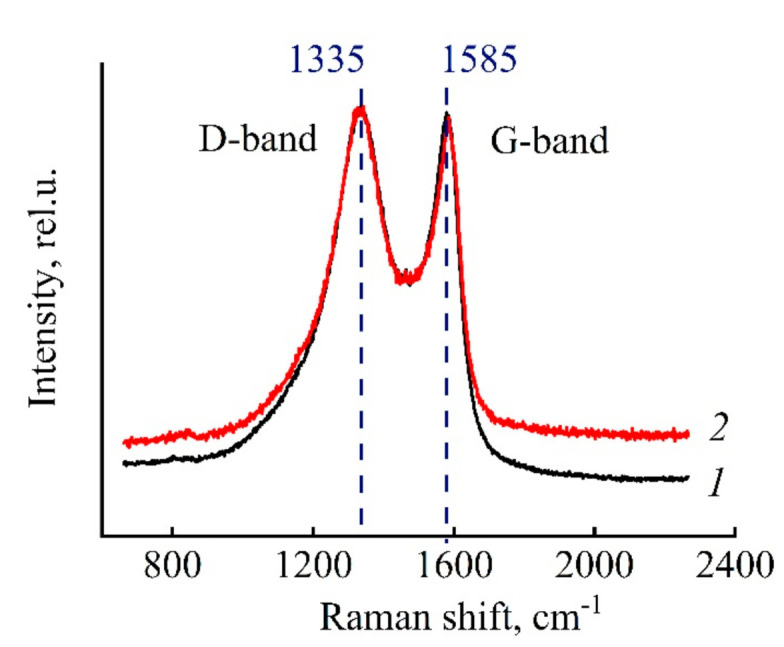
Raman spectra of carbon fibers obtained by heat treatment up to 1200 °C from the Si-0 precursor (*1*) and the composite (Si-10) precursor (*2*).

The Raman spectra (Figure 9) exhibit a broad D band at 1335 cm^−1^ and a G band at 1585 cm^−1^. In other words, the sample contains both disordered (D band) and crystalline carbon G bands. As in the case of X-ray results, the low intensity of the G bands (at the D band level) suggests domination of a disordered (very close to amorphous) structure in the obtained CFs [49,50,51].

Thus, the introduction of BTMSA into the cellulosic matrix influences an order in composite precursor carbon fibers, and its presence enables the ability to increase the carbon yield during their heat treatment. As a result, the obtained CF has mainly an amorphous carbon matrix structure with a small number of ordered regions.

**Figure 10:**
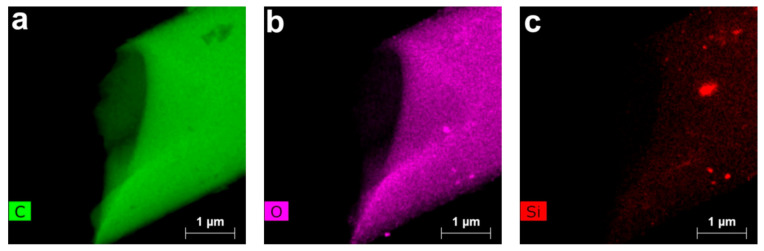
EDS elements mapping of C (**a**), O (**b**), and Si (**c**) for carbon fibers obtained from precursors of Si-10 by heat treatment up to 1200 °C.

The chemical composition and distribution of elements in CF were determined by the EDS (energy-dispersive spectroscopy) method (Figure 10).

Unlike carbon and oxygen, which have a uniform distribution, only the local silicon clusters can be detected on the silicon distribution map; the average size of clusters can reach hundreds of nanometers.

The average mass of silicon in carbon fibers obtained from composite fibers with BTMSA does not exceed tenths of a percent of the total sample mass, which is significantly less compared with the results obtained for precursors with additions of TEOS and VTEOS [31,32,33,34]. It is likely that, in the process of carbonization of precursors with BTMSA, the silicon-containing additive is volatilized, which in turn should also affect the structure of the CF.

## 4. Conclusions

In this work, we prepared composite precursors based on cellulose and BTMSA, studied their structure and properties, and received carbon fibers from them. The addition of BTMSA into the cellulose matrix at the stage of solution preparation makes it possible to obtain composite precursors with a good distribution of silicon in the volume of the polymer. Composite precursors are characterized by a transformation in the structural order with an increase in the content of BTMSA. The mechanical properties of composite fibers obtained are slightly different from those of cellulose fibers, which allows them to be processed into carbon fibers. For all composite fibers, the activation energy of pyrolysis is less than that for cellulose fibers. At the same time, the reaction itself starts first for samples with BTMSA and later for cellulose; i.e., BTMSA reduces the decomposition temperature of cellulose. The type of observed peaks on the DSC curves cardinally changes from the endo peaks intrinsic for cellulose fibers to the combination of endo and exo peaks for composite fibers. This may indicate a number of chemical processes occurring during the decomposition of samples that lead to an increase in the carbon yield at high temperatures. The effect of BTMSA addition on cellulose fibers is also to reduce fiber shrinkage at temperatures above 600 °C. The structure of the obtained fibers differs from the one inherent for high-strength CF, and as a result it is expected to have perspective CTE values.

## Figures and Tables

**Table 1:** Mechanical properties of cellulose and composite fibers.

Sample	Diameter (μm)	Tensile Strength (MPa)	Elongation (%)	Modulus (GPa)
Si-0	17.0 ± 2.8	590 ± 22	11.0 ± 0.8	11.0 ± 0.9
Si-5	12.0 ± 2.3	565 ± 34	7.0 ± 2.1	14.8 ± 2.1
Si-10	12.5 ± 1.5	515 ± 43	7.0 ± 1.2	13.4 ± 2.3

**Table 2:** TG and DTG data for cellulose and composite fibers.

Sample	*T_max_* (°C)	DTG_max_ (% min^−1^)	*T_onset_* (°C)	*E_a_*^1^ (kJ mol^−1^)
Si-0	331	−1.74	281	234
Si-5	328	−2.03	279	162
Si-10	332	−2.14	279	172

^1^*Ea* was calculated using the Broido formula [42].

**Table 3:** TMA data for cellulose and composite fibers.

Sample	(*L*−*L_0_*) ^1^ × 10^3^/*L_0_*, 25–400 °C (step I)	CTE × 10^6^, (average from 30 °C to 400 °C)	(*L*−*L_0_*) × 10^3^/*L_0_*, 25–1200 °C (step II)
Si-0	5.02	13.50	−181.45
Si-5	6.31	16.99	−175.61
Si-10	5.58	14.98	−168.09

^1^*L*—current sample size; *L_0_*—initial sample size.

## Data Availability

The data presented in this study are available on request from the corresponding author.
